# Robert William

**Published:** 1910-09

**Authors:** 


### ROBERT WILLIAM COE, F.R.C.S. Eng.

*Consulting Surgeon to the Bristol General Hospital and to the Bristol Hospital for Sick Children, etc.*

Mr. Coe died at his residence, Pembroke Road, Clifton, on August 11th, 1910, in his 89th year. By his death Bristol has lost its oldest and one of the most distinguished members of the medical profession, also one of the oldest and most respected of its inhabitants. He was born at Bath, and received his general education at a private school. Subsequently he entered as a student at St. George's Hospital, London, passing through the usual courses in the ordinary way without, as far as is known, showing that conspicuous ability which in after-life attained for him that high place among his professional brethren which marked his future career. Mr. Coe obtained the M.R.C.S. in 1844, sixty-six years ago, and the Fellowship by examination in 1852, so that he must have been nearly the oldest Fellow on the roll of the college.

Mr. Coe very early in life settled in Bristol, but then as now practice was slow in coming to very young men; so he determined to proceed to Australia, which in those days was a very remote and unexplored country. He rode on horseback hundreds of miles through the country, and would probably have settled there had not family ties recalled him, when he once more settled in Bristol, laying the foundation of that practice which he retained until within three or four years of his death, and which was of a high-class character. The exact date of Mr. Coe's start in Bristol the writer has not been able to ascertain, but he was elected Surgeon to the Bristol General Hospital in 1852, some years before any member of the existing staff was born. He held the office of Surgeon for twenty-two years, and was elected Consulting Surgeon in 1874. At the time of his death he had held this office during a period of thirty-six years, thus making his official connection with the Hospital a record of fifty-eight years. Mr. Coe was one of the foremost surgeons of his day, a good operator, calm and self-reliant when unforeseen difficulties arose, a man of sound common sense and good judgment, so that his professional brethren frequently sought his advice when surgical consultations were not so common as they are to-day. Mr. Coe was most careful in the treatment of his cases and spent much time in personally attending to the various details of after-treatment. He was a martinet in keeping his dresser and nurses up to the standard of efficiency he required.

Mr. Coe was not a prolific writer on surgical subjects, but his description of the two tedious operations of excision of the wrist and excision of the os calcis cannot be improved on by modern surgeons. But perhaps his classical contribution was a case of ligature of the common carotid for intracranial aneurism following injury; this was believed to be the first case of its kind reported, and it must be remembered that surgery was not so comparatively easy and safe as it now is. It so happened that it fell to the lot of the writer of this notice to have a similar case, in which he followed Mr. Coe's lead with success.

Mr. Coe was connected for many years with the Bristol Medical School in Old Park, and was for many years Lecturer on Surgery there; many of his old pupils still among us have a grateful recollection of their old master.

Mr. Coe was President of the Bath and Bristol Branch of the British Medical Association; he was also President of the Bristol Medico-Chirurgical Society, but in the opening days of his presidential year he had the misfortune to be too unwell to deliver the inaugural address, the only instance in the history of the Society when the address has been omitted. He, together with the present Editor of this *Journal* and the writer of this notice, took a prominent part in the formation of this strictly local medical Society.

Mr. Coe was a man of striking personality, holding strong opinions on most subjects, a keen controversialist, a good fighter, and capable when the occasion arose of expressing himself in vigorous language. These qualities, together with his firmness of purpose, and the care he took to master the details of the case in hand, made him a formidable adversary in any controversy, and were of service when as a member of the preliminary committee for establishing the University College of Bristol, now the Bristol University, he insisted on the claims of the Medical School being duly and amply safe-guarded. Probably few if any of the present Faculty of Medicine are aware how much the position they now hold in the new University is due to his influence in former years. In the early seventies, when the question of the Contagious Diseases Acts was agitating the country, Mr. Coe took a leading part in Bristol, addressing more than one public meeting; and the writer heard him speak in the Victoria Rooms for considerably over an hour against the Acts, arguing in favour of the voluntary submission of affected women to proper treatment in a well-managed hospital, rather than the compulsory examination under the Acts. As a result of Mr. Coe's strenuous advocacy, a Lock Hospital for women was established in Bristol, and he gave much time for many years to the patients of that institution.

Mr. Coe was not the kind of man to remain quiet under a real or supposed grievance. Some of the readers of this *Journal* may remember that in conjunction with Dr. Beddoe he brought before the branch meeting of the British Medical Association what he thought was a just cause of complaint against the authorities of Clifton College on the question of medical attendance on house boys, inasmuch that a boy had been specially sent to Clifton College to be under his care, but he found that he could not attend as his ordinary medical attendant, as it was required that the boy must be under the care of the medical officer of the College, who at that time was the late Dr. Long Fox. Mr. Coe brought forward his case in strong and vigorous language, and feeling ran high, though there was, as usual, much to be said for both sides. Mr. Coe protested that it was not so much the individual wrong he had suffered as the general principle which he thought unjust, and the writer has a vivid recollection of Mr. Coe quoting with emphasis the two lines which Shakespeare puts into the mouth of Hotspur when that character thought he was not getting his proper portion of land:—

> "I'll give thrice so much land to any well-deserving friend,
> But in the way of bargain, mark ye me, I cavil on the ninth part of a hair."

This quotation is reproduced as it is so thoroughly characteristic of the man.

Mr. Coe was married in middle life to a daughter of the late Dr. Borrett, formerly Physician to the Great Yarmouth Hospital. This lady, with a son and daughter, survive him. His eldest son, a gallant and promising young officer of the King's Own Regiment, was killed in the Boer War, at the Battle of Pieter's Hill, to the grief of all who knew him. This loss was the great and abiding sorrow of Mr. Coe's declining years,

PUBLICATIONS.

"Case of Aneurism of Left Internal Caroted within the Cranium," *Assoc. M. J.* 1859.

"On Disease of the Astragalo-calcaneal Joint produced by Injury," *Brit. M. J.* 1863.

"Excision of the Wrist Joint," *Ibid.* 1865.

